# A Deep Ensemble Neural Network with Attention Mechanisms for Lung Abnormality Classification Using Audio Inputs

**DOI:** 10.3390/s22155566

**Published:** 2022-07-26

**Authors:** Conor Wall, Li Zhang, Yonghong Yu, Akshi Kumar, Rong Gao

**Affiliations:** 1 Department of Computer and Information Sciences, Faculty of Engineering and Environment, University of Northumbria, Newcastle upon Tyne NE1 8ST, UK; cwall1996@hotmail.com; 2 Department of Computer Science, Royal Holloway University of London, Egham TW20 0EX, UK; 3 College of Tongda, Nanjing University of Posts and Telecommunications, Nanjing 210049, China; yuyh@njupt.edu.cn; 4 Department of Computing and Mathematics, Manchester Metropolitan University, Manchester M15 6BH, UK; akshi.kumar@mmu.ac.uk; 5 School of Computer Science, Hubei University of Technology, Wuhan 430068, China; gaorong@hbut.edu.cn

**Keywords:** Long Short-Term Memory, Gated Recurrent Unit, bidirectional Recurrent Neural Network, Convolutional Neural Network, attention mechanism, ensemble model, audio lung abnormality classification

## Abstract

Medical audio classification for lung abnormality diagnosis is a challenging problem owing to comparatively unstructured audio signals present in the respiratory sound clips. To tackle such challenges, we propose an ensemble model by incorporating diverse deep neural networks with attention mechanisms for undertaking lung abnormality and COVID-19 diagnosis using respiratory, speech, and coughing audio inputs. Specifically, four base deep networks are proposed, which include attention-based Convolutional Recurrent Neural Network (A-CRNN), attention-based bidirectional Long Short-Term Memory (A-BiLSTM), attention-based bidirectional Gated Recurrent Unit (A-BiGRU), as well as Convolutional Neural Network (CNN). A Particle Swarm Optimization (PSO) algorithm is used to optimize the training parameters of each network. An ensemble mechanism is used to integrate the outputs of these base networks by averaging the probability predictions of each class. Evaluated using respiratory ICBHI, Coswara breathing, speech, and cough datasets, as well as a combination of ICBHI and Coswara breathing databases, our ensemble model and base networks achieve ICBHI scores ranging from 0.920 to 0.9766. Most importantly, the empirical results indicate that a positive COVID-19 diagnosis can be distinguished to a high degree from other more common respiratory diseases using audio recordings, based on the combined ICBHI and Coswara breathing datasets.

## 1. Introduction

Deep learning has emerged as one of the most popular techniques for video and signal processing tasks, owing to its recent breakthrough and advancement [1]. A significant number of deep architectures have been proposed for medical diagnosis with respect to diabetic retinopathy screening, brain tumor, and leukemia diagnosis. Deep Neural Networks have also shown significantly improved performance for medical sound classification [2]. As an example, Convolutional Neural Networks (CNNs) have been used for the classification of cardiovascular phonocardiograms (PCG) pertaining to heart abnormality identification, based on the extraction of Mel-frequency cepstral coefficients (MFCC) features [3]. Such deep networks showed much enhanced capabilities for heart abnormality classification in comparison with traditional machine learning methods such as Support Vector Machine (SVM) and Multilayer Perceptron (MLP). Recurrent Neural Networks (RNNs) have also shown effectiveness for temporal feature extraction with respect to language generation and audio classification. As two popular types of RNNs, Long Short-Term Memory (LSTM) and Gated Recurrent Unit (GRU) have been widely adopted in signal classification and time series forecasting [1,4]. These networks are similar in functionality, with the primary difference being that the GRU combines the “forget” and “input” gates into an “update” gate, as well as having a “reset” gate, instead of an “output” gate as in LSTM. This difference in architecture results in the GRU model having a simpler topology with fewer parameters than those of an LSTM unit [5]. Both LSTM and GRU have been used in recent studies for medical audio classification. For instance, Kochetov et al. [4] proposed a Noise-Masking Recurrent Neural Network (NMRNN) for respiratory abnormality classification based on the MFCC features. Besides using both LSTM and GRU networks, their model applied a noise classifier to distinguish and eliminate any redundant noise in the audio files, simultaneously. Their work was evaluated using the ICBHI dataset [6] for the classification of normal, wheeze, crackle, and both crackle and wheeze cases. A GRU network was also exploited for audio scene classification in [7]. Their findings indicated the growing potential of using GRU networks for audio classification, owing to the fact that the network achieved an F1-score of 97.7% for the classification of 19 scenes using the LITIS-Rouen dataset.

Motivated by the above studies, in this research, we employ LSTM and GRU networks for the identification of diverse lung diseases and COVID-19 conditions using respiratory, coughing, and speech datasets. In addition, we employ five medical audio datasets in this research, i.e., ICBHI [6], Coswara breathing [8], Coswara speech [8], Coswara coughing [8], and the combination of ICBHI and Coswara breathing, to distinguish between different types of commonly seen lung diseases as well as between such lung conditions and COVID-19. Specifically, the ICBHI dataset contains audio respiratory recordings of six lung conditions, i.e., healthy, chronic obstructive pulmonary disease (COPD), pneumonia, bronchiectasis, bronchiolitis, and upper respiratory tract infection (URTI). The Coswara dataset contains coughing, speech, and breathing audio samples associated with COVID-19 conditions. In other words, these Coswara datasets can be used to identify positive and negative COVID-19 cases through coughing, speech, and respiratory recordings, respectively. In addition, we also combine ICBHI and Coswara breathing datasets to distinguish COVID-19 from other respiratory diseases (e.g., COPD and pneumonia) based on breathing clips.

However, owing to the intermit characteristics of respiratory/coughing sound recordings, different recording mechanisms, and background and white noise, medical audio classification performance can be hindered using the above datasets. In addition, for the Coswara speech dataset, a range of accents and dialects are also present, which could affect network performance with respect to positive and negative COVID-19 classification through speech alone.

**Figure 1:**
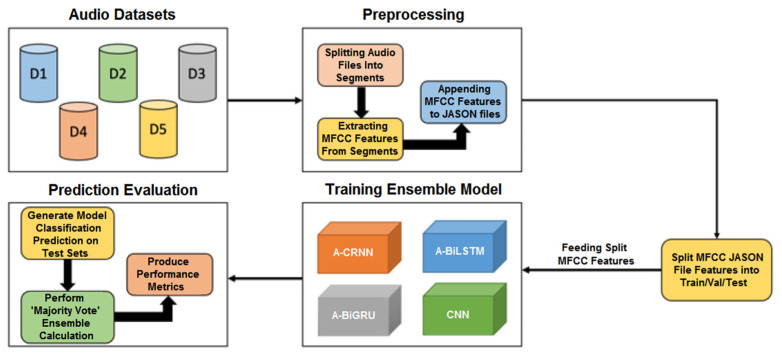
The proposed ensemble model comprising four base networks for diverse lung abnormality and COVID-19 diagnosis.

To tackle the above challenges and ascertain a reliable diagnosis, we propose a set of deep networks, i.e., 1D CNN, attention-based Convolutional Recurrent Neural Network (A-CRNN), attention-based bidirectional LSTM (A-BiLSTM), and attention-based bidirectional GRU (A-BiGRU), as well as an ensemble model embedding the above networks for classifying a variety of lung conditions. Evolutionary algorithms such as Particle Swarm Optimization (PSO) are also used to identify the optimal hyper-parameters of the above base deep networks. The ensemble model incorporates various base networks with distinctive learning behaviors and hyper-parameter settings to increase model diversity. Figure 1 shows the proposed system dataflow. The contributions of this research are summarized as follows.

- We propose a PSO-based evolving ensemble model for lung abnormality classification, which integrates four types of deep networks, i.e., A-CRNN, A-BiLSTM, A-BiGRU, and 1D CNN, with the attempt to generate diverse discriminative acoustic representations to enhance classification performance. Specifically, the A-CRNN model comprises 1D convolutional and BiLSTM layers to diversify feature learning mechanisms, while A-BiLSTM exploits bidirectional LSTM layers to learn feature representations from both forward and backward directions. A-BiGRU adopts similar bidirectional RNN layers (i.e., BiGRU layers) but with different gating mechanisms to explore network feature learning capabilities in tackling disease diagnosis. A 1D CNN model is also proposed which embeds a set of 1D convolutional layers with scalar multiplication and addition operations for extracting sequential temporal cues. On top of this, attention mechanisms are also exploited in A-CRNN, A-BiLSTM, and A-BiGRU for extracting more discriminative signal dynamics.
- To maximize network performance and diversify model learning behaviors, a PSO model is used to optimize the learning rate, batch size, and the number of training epochs for A-CRNN, A-BiLSTM, A-BiGRU, and CNN. The devised networks with distinctive learning configurations illustrate more diversified learning behaviors to enhance ensemble model robustness. The resulting ensemble model utilizes an average probability method to integrate the outputs of these optimized base networks.
- Evaluated using several challenging medical sound datasets, the proposed ensemble model outperforms existing methods for abnormal respiratory, coughing, and speech sound classification with respect to diverse lung disease and COVID-19 cases. In particular, the proposed base and ensemble models show great efficiency in distinguishing common respiratory diseases from COVID-19 using respiratory audio clips. To the best of our knowledge, we are also the first study to explore the classification of COVID-19 against diverse other commonly seen lung conditions.

The paper is organized as follows. Section 2 presents state-of-the-art existing studies for audio classification. The proposed audio classification networks with optimal hyper-parameter selection as well as ensemble model construction are depicted in Section 3. A comprehensive evaluation is presented in Section 4. We conclude this research and identify future directions in Section 5. 

## 2. Related Work

### 2.1. General Audio Classification

There has been a wide range of studies using deep learning techniques for audio classification. As an example, Choi et al. [9] proposed a Convolutional Recurrent Neural Network (CRNN) for music classification. Their work described how the CRNN model was used to train on the Million Song dataset, which consisted of numerous song clips from categories such as genre, mood, era, and music instrument [10]. As efficient assets for classifying audio datasets, MFCC features were firstly extracted from the dataset using the python package Librosa [11]. The MFCC features presented the logarithmic measure of the Mel magnitude spectrum and contained sufficient discriminating properties. Their studies indicated that the CRNN model, which consisted of four conv2d layers and two RNN layers, performed better than the three baseline CNN models, with the only downside being the higher number of parameters, leading to higher computational costs. 

Chen and Li [12] proposed a multi-feature combined network by combining two streams, i.e., CNN-BiLSTM and 1D DNN, for song emotion classification using audio inputs. Another 1D network with several fully connected layers was also used to detect emotions in lyrics text inputs. An ensemble stacking method was used to combine the emotion detection outputs from both audio and text inputs. The Million Song dataset consisting of 500 music samples with each of the 4 emotion classes: anger, sadness, relaxation, and happiness, was used to investigate model efficiency. Their hybrid network obtained an average accuracy rate of 68% for classifying four emotion classes, whereas the three baseline models, i.e., CNN-LSTM, CNN, and LSTM, obtained accuracy rates of 63%, 59%, and 50%, respectively. This ascertained that using a BiLSTM layer may have a strong positive influence on improving classification performance, making it a worthy addition to the experimental studies in this research.

### 2.2. Related Studies Using the ICBHI Dataset

Perna and Tagarelli [13] implemented RNN models (i.e., BiLSTM, LSTM, and GRU) to train and classify the respiratory dataset, i.e., ICBHI, on both pathology and anomaly levels. The pathology-level classification includes two different tasks, i.e., the binary classification of healthy and unhealthy cases, and one ternary classification of healthy, chronic, or non-chronic conditions. On the other hand, the four-class-driven anomaly-level prediction focused on the identification of normal, wheeze, crackle, and the presence of both conditions. Their work employed BiLSTM, LSTM, and GRU networks, where seven different model configurations were considered. These configurations differed by several experimental settings, such as window size and step, frame size, and extracted audio features. The results from the study showed several notable observations. The first observation was that for the four-class anomaly-driven prediction, all seven settings had a similar performance with respect to each network, with the ICBHI scores ranging from 0.71 to 0.74, while for the pathology-driven predictions, the ICBHI scores ranged from 0.86 to 0.91. This indicated the strong and robust performance of the RNN models, regardless of the configuration settings chosen. In particular, their experiments demonstrated that the LSTM model performed the strongest over the range of experiments, while the GRU model generally performed the worst. Additionally, the BiLSTM model performed very inconsistently, having the strongest results for some of the experiments, while also having the weakest results for others, as well as being the most computationally intensive.

### 2.3. Related Studies Using the Coswara Dataset

Concerning the Coswara dataset, although the dataset is new and the audio samples in the dataset are recorded from mobile devices, there are several studies that demonstrate strong classification performance using different machine learning methods. For instance, Pahar et al. [14] demonstrated the use of both the Coswara dataset and the SARS-CoV-2 South Africa (Sarcos) dataset. Their work adopted a range of machine learning models, such as Resnet50, LSTM, CNN, MLP, SVM, and logistic regression (LR), for the classification of different lung abnormalities. MFCC features were also adopted in their studies. Two evaluation strategies were employed in their experiments, one being both trained and tested on the Coswara dataset, and the other being trained on the Coswara dataset and tested on the Sarcos dataset, with the attempt to determine if the data in the Coswara dataset would be suitable for classification on audio files outside of its specific recording conditions. The classes for both datasets were coughing recordings from subjects with either a positive or a negative diagnosis for COVID-19. For the first set of evaluations where the Coswara dataset was used for training and testing, it was found that Resnet50 and LSTM recorded the highest accuracy performances, with accuracy rates of at least 93.65% and 95.3%, respectively. Meanwhile, the MLP, LR, and SVM all performed significantly worse. For the second set of evaluations, where the Coswara and Sarcos datasets were employed for training and testing, respectively, the empirical results indicated that LSTM in conjunction with the greedy search algorithm, i.e., Sequential Forward Selection (SFS) [15], was the best-performing network, with an accuracy rate of 92.91% on the test set, whereas other baseline models demonstrated results of 73.02–74.58%. As revealed by both studies, the most performant model for audio classification on the Coswara dataset was the LSTM network.

Muguli et al. [16] presented a challenge in which 29 teams performed binary audio classification on the Coswara dataset, i.e., using subjects who have a positive or a negative COVID-19 diagnosis. Unlike the previous works, this study featured two tracks, Track-1 being the primary track, focusing on cough sounds, and Track-2, focusing on a collection of breathing and speech sounds, with the latter including vowel phonation and number counting recordings. A range of feature extraction choices were made across the 29 teams, including MFCC, spectrograms, and feature embeddings. Similarly, there was also a range of model choices including comparatively more traditional methods such as an LR, MLP, and Random Forest (RF), as well as state-of-the-art deep networks such as CNN (e.g., ResNet) and LSTM models. Following the 22-day period of the challenge, it was found that for the primary Track-1, the best team showcased an AUC of 87.07%, which far outperformed the baseline RF model with an AUC of 70.63%, for classifying positive/negative COVID-19 conditions. All the aforementioned studies indicate that there is vast potential for developing a high-performing model for the use of audio classification on both the ICBHI and Coswara datasets.

### 2.4. Attention Mechanisms

A notable state-of-the-art addition to the investigation in this research is the attention mechanism, which has been extensively examined in numerous studies as part of RNN architectures. Such attention schemes have demonstrated promising results in areas such as speech recognition, natural language processing (NLP), and image description generation. The attention mechanism has the adaptive capacity to learn the relationship between each of the input features over numerous time steps to predict the current timeframe [17,18].

Zhang et al. [18] introduced a CRNN architecture incorporating an attention mechanism, with the attempt to identify diverse environmental sound signals. Their model was tested using the ambient audio datasets, i.e., ESC-50 and ESC-10, with 50 and 10 environmental sound categories, respectively [19]. According to the empirical results from the study, the attention mechanism produced a considerable boost in accuracy, with an average increase of more than 2% for both environmental sound datasets. The potential boost in classification accuracy, and the ability to determine if such a boost exists, are the two primary reasons why the attention mechanism has been chosen to be included for the investigation in our research studies.

Moreover, Wall et al. [20] utilized BiLSTM and BiGRU with attention mechanisms for lung abnormality classification using the ICBHI and Coswara cough datasets. Sait et al. [21] employed transfer learning based on Inception-v3 combined with MLP for COVID-19 diagnosis using breathing and chest X-ray image inputs, while Wall et al. [22] and Perna [23] studied BiLSTM and CNN for common lung abnormality diagnosis using breathing audio inputs, respectively. García-Ordás et al. [24] studied a 2D CNN in combination with data augmentation and oversampling techniques, such as Variational Convolutional Autoencoder, for respiratory abnormality classification.

Besides the above, a variety of other existing studies have also been exploited for undertaking respiratory, heart, and environmental sound classification. For example, Boddapati et al. [25] conducted environmental sound identification using AlexNet and GoogLeNet. Zhang et al. [26] exploited an ensemble of CRNN models with hyper-parameter selection for respiratory, heart, and environmental sound classification, while 1D CNNs were utilized by Li et al. [27] and Xiao et al. [28] for heart abnormality classification using audio inputs. We summarize the aforementioned studies in Table 1. In comparison with these existing studies, we propose an evolving ensemble model integrating four different types of CNN and RNN networks (i.e., CRNN, BiLSTM, BiGRU, and 1D CNN) with attention mechanisms and PSO-based hyper-parameter identification to generate diverse discriminative acoustic representations and increase model robustness.

### 2.5. Particle Swarm Optimization

PSO is a widely exploited population-based heuristic optimization algorithm inspired by the bird flocking and fish schooling. PSO has illustrated great capabilities in solving global optimization problems owing to its effective search strategies, model scalability, and robustness [30,31]. This enables the PSO algorithm to be highly suitable for the purpose of optimizing hyper-parameters of deep learning models, e.g., learning rate, momentum, and batch size. Existing studies [32] showed a comparison of PSO and a grid search, for optimizing the number of hidden layers and the number of neurons in each hidden layer in several shallow and deep architectures. Their studies indicated that not only did the PSO algorithm obtain superior classification performance but it also decreased training times by 77–85%, in comparison with those of the grid search algorithm.

Improving classification results and decreasing training costs [33] are the two primary reasons for the inclusion of the PSO algorithm in this research. In particular, the PSO algorithm is used for optimizing hyper-parameters, i.e., the learning rate, batch size, and the number of training epochs, when training each base deep network in the ensemble model. We introduce each proposed deep network with attention mechanisms and the resulting ensemble model in detail below.

## 3. Methodology

In this research, we propose an ensemble model comprising four base deep networks for various lung abnormality and COVID-19 diagnoses. Firstly, four base deep networks were proposed, i.e., A-CRNN, A-BiLSTM, A-BiGRU, and 1D CNN. A PSO algorithm was used to optimize the learning rate, batch size, and the number of training epochs of each of the above networks to improve performance. The yielded optimized settings were used to train the respective base networks. The mean result of the prediction probabilities produced by the base networks was subsequently calculated and used to determine the final prediction in the ensemble model. Evaluated using the five audio datasets, the proposed ensemble model showed reliable performances in comparison with those of existing studies for diverse lung abnormality and COVID-19 diagnoses using respiratory, speech, and cough audio inputs. We first introduce the construction of several customized datasets in our studies.

### 3.1. Dataset Preprocessing

As previously mentioned, the two datasets employed in our experimental studies were ICBHI and Coswara. However, we decided to utilize these two databases to create five customized datasets, labeled as D1–D5. D1 is the standalone ICBHI dataset, D2 is a Coswara dataset consisting of only coughing sounds, D3 is a Coswara dataset consisting of only speech sounds, D4 is a Coswara dataset consisting of only breathing sounds, and D5 is a combination of the ICBHI and Coswara breathing sounds datasets. 

In particular, for the combined dataset D5, one major aspect is that as the recordings from the ICBHI dataset are of breathing sounds, any COVID-19 recordings used must also contain breathing sounds of the same nature, as built in this study by using the Coswara breathing dataset. It is also crucial that sufficient recordings are provided, as well as being balanced with the other classes included.

In particular, with respect to using the Coswara cough, speech, and breathing datasets for COVID-19 diagnosis, we have decided that these would be the suitable evaluation strategies for the following reasons. One reason is that classifying respiratory diseases on a range of sounds, i.e., speech, breathing, and coughing, would be beneficial for the primary reason of determining the most effective channel for respiratory disease diagnosis. It may also be used as a reference to advise future audio sample collection for dataset construction and audio classification.

In addition, the creation of a separate dataset (D5) that consists of ICBHI and Coswara breathing sounds clips would allow for distinguishing a range of respiratory diseases from a positive COVID-19 diagnosis. To the best of our knowledge, we are the first to exploit the diagnosis of COVID-19 against other common lung conditions.

Table 2 outlines the contents of each of the five datasets, including the respiratory disease classes and number of audio files within each of the classes. 

#### 3.1.1. Pre-Processing for the ICBHI Dataset (D1)

The ICBHI dataset [6] has 920 annotated respiratory recordings from 126 subjects. For each subject, different chest positions are used for the recording of respiratory audio clips. The dataset provides samples with the following respiratory diseases, i.e., COPD (793), lower respiratory tract infection (LRTI) (2), URTI (23), asthma (1), bronchiectasis (16), bronchiolitis (13), pneumonia (37), and healthy (35) cases. Although the ICBHI dataset originally includes eight classes, there are only one and two audio samples for asthma and LRTI classes, respectively. Therefore, they were not used in our experiments. We employed an 80–20 subject-independent train–test split in our experiments by mainly following the official train–test split provided by the ICBHI dataset. To be specific, in our experiments, the samples from training and test sets are subject-independent, i.e., there is no overlapping of the subjects in the training and test sets. We followed the official train and test splits for all the classes except for COPD to form the training and test sets. For COPD, the official split uses a 56–44 subject-independent train–test split. We moved some test samples to the training set but still maintained a subject-independent division for this class. In this way, we achieved an 80–20 subject-independent train–test split for the overall dataset. Table 3 illustrates the detailed training and test sample sizes used in our experiments.

As indicated in Table 3, the training sample size (i.e., 648) of COPD is comparatively much larger than those of other classes. Thus, the training set is severely imbalanced. To tackle such problems, audio augmentation techniques such as noise addition and pitch and time shifting have been performed, but as indicated in the existing studies [24,34], such low-level augmentation strategies alone are not able to make a significant impact on the classification boundaries, owing to the unstructured challenging nature of such respiratory audio data. As recommended by existing studies [34], data duplication has been performed to increase sample sizes of the minority classes. For example, we duplicated the 30 training instances for pneumonia 22 times to generate a new training set of 660 recordings. A similar case was applied to other classes. Such strategies were used in conjunction with audio augmentation operations to strengthen respiratory audio signal patterns (especially for minority classes) and balance data distribution. Moreover, the above data duplication and augmentation methods were only applied for the training set, while the test set remained intact. The second column in Table 3 shows the augmented training sizes for different classes. Overall, a total of 3996 audios were used for training, with the unseen 184 clips contributed by different subjects for testing. 

#### 3.1.2. Pre-Processing for the Coswara Cough, Speech, and Breathing Datasets

With respect to Coswara cough (D2), speech (D3), and breathing (D4) datasets, as seen in Table 2, balanced positive and negative sample sizes were extracted to safeguard against any potential issues arising due to imbalanced sample distributions. In our experiments, a random 80–20 split was used to divide the training and test sets for each class in each dataset.

#### 3.1.3. Pre-Processing for the Combined Dataset (D5) Based on ICBHI and Coswara Breathing Databases 

To further test model efficiency, we constructed the combined dataset D5 by integrating the ICBHI and Coswara breathing datasets, with the attempt to classify COVID-19 against a number of other lung abnormalities. We used all the positive COVID-19 samples, i.e., 101 clips, from D4 to combine with D1 (the ICBHI dataset). Since the ICBHI dataset uses a subject-independent split, we also used an 80–20 subject-independent split for the positive COVID-19 class instances from D4, where 80% of the samples (i.e., 81 clips) were used for training and 20% of the samples (i.e., 20 recordings) from unseen subjects were used for testing. 

As mentioned earlier, as indicated in Table 3, ICBHI is severely imbalanced across all the disease cases, with COPD having the largest dominating training sample size (i.e., 648). To balance class distributions, we duplicated the 81 positive training clips from D4 8 times to yield 648 training instances for the positive COVID-19 class. Each network was trained using these augmented positive COVID-19 cases along with the augmented training samples from the ICBHI dataset for six other lung conditions. The training set of the combined dataset D5 is illustrated in Table 4. Again, the data duplication and augmentation procedures were only utilized for the training set. The unseen test set contributed by different subjects with respect to COVID-19 and six other respiratory conditions remained unchanged and was used for testing.

### 3.2. Feature Extraction

We subsequently elaborated feature extraction from audio signals. As in existing studies, we also extracted MFCC features from audio inputs for lung abnormality identification. As previously stated, MFCC properties possess sufficient discriminative characteristics for describing audio signals, leading to a high classification performance of numerous audio recognition tasks. In our experiment, MFCC features were extracted using the python package Librosa. 

During the pre-processing step, numerous factors must be considered. The first step is to decide how to ensure the model has adequate input features. We addressed this issue by segmenting audio samples. Specifically, each audio input was divided into segments based on the sample rate and duration of the audio clip.

After splitting the audio clips into segments, all the MFCC features from each segment were retrieved and appended to a dictionary with their class label. Factors, such as the parameters for Fast Fourier Transform (FFT) and hop length, must be determined to generate the MFCC features. 

The FFT algorithm is typically used to transform a signal from its native domain, which in this case is time, to a target frequency domain representation. In the context of MFCC, FFT is applied to each frame to determine the frequency spectrum. This was achieved by using a technique known as the Short-Time Fourier-Transform (STFT), from which the power spectrum was generated. 

After calculating the power spectrum, triangular filters on the Mel-scale were applied to the power spectrum to extract frequency bands. Next, using these frequency bands, the Mel frequency was calculated using Equation (1) [18]:

(1) $$\mathrm{Mel}\left(f\right)=1127\times \ln\left(1+\frac{f}{700}\right)$$

The formula in Equation (1) converts the audio input to the Mel frequency in hertz, i.e., Mel(f). Specifically, it is calculated by multiplying 1127 with the natural logarithm (ln), where a constant value of 1 plus the frequency in hertz(f) divided by the corner frequency of 700 is used.

The hop length values, together with the FFT algorithm, determine how many frames are taken from each segment. The default values used are 2048 for FFT and 512 for hop length, which were utilized for this study. Following the extraction of the MFCC feature from the audio recordings, each was added to a JSON file, which served as the input file for training the model architectures. 

Once the JSON file consisting of the MFCC features was generated, the features were then split into training, validation, and testing sets based on the settings of each experiment. The use of a validation dataset is predominantly for the purpose of helping ensure that the training process is as robust as possible, as well as providing the ability to tune the hyper-parameters more accurately. 

### 3.3. The Proposed Models

#### 3.3.1. A-CRNN

The first proposed model in this research is a CRNN with an attention mechanism (A-CRNN). It incorporates a variety of layers, such as 1D convolutional and max pooling layers, bidirectional and unidirectional LSTM layers, dense layers, and an attention layer. Table 5 outlines the specific layer architecture with respect to the A-CRNN model.

#### 3.3.2. LSTM

The second exploited architecture is the BiLSTM network with attention mechanism (A-BiLSTM). This model is composed of bidirectional and unidirectional LSTM layers, dense layers, and an attention layer. 

Moreover, a BiLSTM layer contains two RNN layers of the same kind, such as two LSTM layers. These two layers ensure that input features may be processed in both forward and backward time series. This allows the model to better determine the relationships between components in the input sequence by using information in both forward and backward directions [35,36]. Therefore, BiLSTM layers were adopted in our network. In addition, the regularization parameter (i.e., weight decay) and a dropout layer were also determined in trial-and-error in this model. The primary reason for selecting optimal settings of these parameters is owing to the efficiency of such techniques in reducing the amount of overfitting that may occur during the training of neural networks [37].

Moreover, to determine if the convolutional layers used in the aforementioned A-CRNN model have a positive effect on performance, the A-BiLSTM model does not implement such CNN layers. This will be used as means of comparison. Table 6 below outlines the specific layer architecture for the A-BiLSTM model.

#### 3.3.3. A-BiGRU

The third employed network is the BiGRU model with an attention mechanism (A-BiGRU). This model is similar in architecture to the A-BiLSTM network, with the only differing aspect being the implementation of bidirectional and unidirectional GRU layers in place of the bidirectional and unidirectional LSTM layers. Table 7 outlines the specific architecture for the A-BiGRU network.

#### 3.3.4. CNN

The fourth model adopted in this research is a CNN. This model is the most unique of the four models, using only a 1D convolutional layer, as opposed to the other models which incorporate RNN layers in combination with attention mechanisms. Table 8 shows the CNN architecture.

#### 3.3.5. PSO-Based Hyper-Parameter Selection

We conducted optimal hyper-parameter selection using the PSO model to optimize the learning configurations of each network. Such optimized settings equip the network with distinctive learning behaviors and help prevent the network from overfitting. In addition, the PSO algorithm is widely adopted for solving diverse optimization problems, such as ensemble classifier reduction [38], feature selection [39], deep architecture generation [40], hyper-parameter identification [41], and job scheduling [42]. In comparison with other swarm intelligence algorithms, such as the Firefly Algorithm and Simulated Annealing, it searches for the most optimal solution by following both personal and global best experiences. The search process of PSO is defined in Equations (2) and (3):

(2) $$v_{id}^{t+1}=w\times v_{id}^{t}+c_{1}\times r_{1}\times \left(p_{id}-x_{id}^{t}\right)+c_{2}\times r_{2}\times \left(p_{gd}-x_{id}^{t}\right)$$

(3) $$x_{id}^{t+1}=x_{id}^{t}+v_{id}^{t+1}$$

where $x_{id}^{t+1}$ and $v_{id}^{t+1}$ represent the position and velocity of the i-th particle in the d-th dimension and the t+1-th iteration, respectively, with w as the inertia weight which adjusts the contribution of the previous velocity, $v_{id}^{t}$. $c_{1}$ and $c_{2}$ are acceleration coefficients which determine the influence of the cognitive and social components, with $p_{id}$ and $p_{gd}$ denoting the personal and global best solutions, respectively. Moreover, $r_{1}$ and $r_{2}$ are random coefficients, with each element randomly generated in the range of [0, 1]. As indicated in Equation (2), the search process of the PSO algorithm is guided by the personal and global best solutions, simultaneously. In other words, each particle learns from both personal and global best experiences to balance between diversification and intensification. We adopted the PSO algorithm for hyper-parameter identification for each proposed network to increase network robustness and diversity.

Specifically, the search of the optimal learning configurations was conducted as follows. Firstly, a swarm was initialized with random particle positions. Each particle position has three dimensions which represents the three optimized elements, i.e., the learning rate, batch size, and maximum number of epochs. Such a configuration embedded in each particle was used to setup each network training option. The fitness of the particle is obtained using the accuracy rate of the validation set. The PSO algorithm employs the personal and global best solutions to guide the search of optimal hyper-parameter configurations. The most optimal three hyper-parameters recommended by the global best solution were used to setup the final network. The devised network was trained using the combined training and validation sets and tested using the unseen instances in the test set. We adopted the following experimental settings for PSO-based optimal hyper-parameter selection, i.e., dimension = 3, trial = 10, and a maximum number of function evaluations (population × a maximum number of iterations) = 450. Such a setting was applied for optimizing hyper-parameters for each base network. The optimized networks were subsequently used to construct the ensemble model, where a mean probability method was exploited to incorporate all the results from the base networks.

#### 3.3.6. The Ensemble Model

After the construction of the four optimized base networks, we subsequently developed an ensemble model that incorporates the four proposed base networks. 

The ensemble strategy is a broad term to describe methods that combine multiple base learners to generate a joint decision [43]. These learners can be any types of classification algorithms. The ensemble learning scheme is to ensure that any errors from any single learner would then be compensated by the other learners as part of the ensemble, which overall would theoretically lead to a stronger classification performance [44].

There are several ensemble learning approaches, such as majority voting, bagging, and stacking [45]. In this research, we employed the majority voting strategy. The majority voting approach is one of the simplest and most effective methods to implement, of which there are two implementations, namely ‘hard’ and ‘soft’.

The ‘soft approach’ was the method chosen for this investigation and involves determining which prediction to make by looking at the predictions made by each classifier and calculating the average probability across each class. The class with the highest average probability across the classifiers is then the ensemble prediction decision [46]. 

Overall, from the inspection of the aforementioned model architectures in Table 5, Table 6, Table 7 and Table 8, various state-of-the-art deep learning techniques have been incorporated in the proposed base networks. Each of the four models adopts separate unique neural network implementations, with the main components, i.e., LSTM, GRU, and CNN layers, all showing increasing promise in recent literature for audio classification proficiency. We subsequently introduce the construction of the ensemble model for audio classification.

##### Ensemble Model Training

As each base model consists of different deep learning techniques, it was imperative that during the training process, there was a level of flexibility for model training to ensure no overfitting occurred. This meant several precautions were required to be implemented to achieve this. 

The first of which was the callback technique, i.e., early stopping. Early stopping is a validation method that can be used to determine when overfitting is starting to occur while the model is training. This can be carried out through the inclusion of a validation set and a validation metric, of which there are several that can be chosen [47]. The validation metric used in this research is the validation loss, which is calculated during the training process for each epoch. A ‘patience’ setting is also needed, which is a value used with the early stopping method to analyze whether validation loss has decreased within the said value of epochs. If the validation loss has not decreased within a predefined number of epochs, then the training will stop. This ultimately helps prevent any overfitting that may occur during the training process. We set the ‘patience’ value to 5 epochs for each of the models during the training for each dataset. Other hyper-parameters such as weight decay and recurrent dropout layers were also identified through trial-and-error to prevent the network from learning too closely from the training set to avoid overfitting.

The second strategy used for model training to avoid overfitting and optimize performance was to create different hyper-parameter settings for each separate model in the ensemble. As each model has a different number of parameters, as well as its own unique learning mechanism, ensuring that each model has its own optimal hyper-parameter settings was imperative. Therefore, the identification of optimal learning hyper-parameters (i.e., the learning rate, batch size, and the maximum number of learning epochs) was also conducted using the PSO model to increase model robustness and avoid overfitting.

The determination of the hyper-parameter settings, i.e., the learning rate, training epoch, and batch size, could potentially be found through an optimization method such as a grid search. However, such an exhaustive search process is time-consuming. In some cases, it is even infeasible. Therefore, in this research, we employed PSO for hyper-parameter fine-tuning. The following settings were used for hyper-parameter selection, i.e., population size = 15, dimension = 3, and a maximum number of iterations = 30. We performed 10 trials for optimizing each network. The detailed identified mean hyper-parameters for each base network over 10 runs are presented in Section 4.

## 4. Evaluation

To test model efficiency, we employed the five generated datasets for performance comparison. Following the extensive and rigorous training process, results were recorded from each of the experiments for the proposed base and ensemble networks. 

The most proficient hyper-parameters identified using the PSO algorithm during the training stage for all the five datasets are illustrated in Table 9, Table 10, Table 11, Table 12 and Table 13. They were used to setup each optimized network, which were subsequently trained using the training set and tested using the unseen audio clips in the test set.

Regarding medical diagnoses, the metrics such as sensitivity and specificity are widely used as a standard for measuring the performance of a diagnostic method, with sensitivity referring to the rate of true positive, and specificity referring to the rate of true negative [48]. 

One reason for selecting sensitivity and specificity is that using a simple test accuracy metric can be misleading, as a high test accuracy could potentially still include a high percentage of false positives and false negatives, indicating that the method of diagnosis is less useful than the high test accuracy implies.

In this research, we used four metrics, i.e., sensitivity, specificity, the ICBHI score, and accuracy rate, for performance comparison using the five datasets, where the ICBHI score refers to an average of the sensitivity and specificity results.

The ICBHI score metric has previously been used in studies regarding the lung and heart abnormality detection [13,27]. Its inclusion in this study was to both compare the ensemble results with these related studies regarding the ICBHI dataset, and to provide a metric for comparing the results from the Coswara datasets, as well as the combined Coswara and ICBHI dataset. We discuss the detailed evaluation results using D1–D5 below.

### 4.1. Evaluation Results for D1 (ICBHI) Using a Subject-Independent Split

As discussed earlier, we used an 80–20 subject-independent train–test split for the evaluation of the ICBHI dataset. Results of the optimized base and ensemble models for the ICBHI dataset using the subject-independent split are illustrated in Table 14. The optimized A-CRNN model achieved the best ICBHI score and accuracy rate, followed by those of A-BiLSTM and CNN, with A-BiGRU as the least performant network. The devised A-CRNN model with optimized learning settings showed significant capabilities in extracting distinctive temporal dynamics using both 1D convolutional and BiLSTM layers with attention mechanisms, whereas other networks such as CNN, A-BiLSTM, and A-BiGRU employed either 1D convolutional or BiLSTM/BiGRU layers for sequential feature extraction, resulting in less efficient audio representations. The resulting ensemble model integrating these different types of optimized networks with different learning behaviors embeds sufficient diversity and complementary properties to further improve base model performance. Table 15 illustrates the detailed confusion matrix of our devised ensemble network. 

### 4.2. Evaluation Results for Coswara Cough (D2), Speech (D3), and Breathing (D4) Datasets Using Random Splits

We subsequently evaluated the Coswara cough (D2), speech (D3), and breathing (D4) datasets. To compare with existing studies [20], a random 80–20 split was performed for each of these Coswara datasets for model evaluation. Table 16, Table 17, Table 18, Table 19, Table 20 and Table 21 show the detailed evaluation results and the confusion matrices of the ensemble model for the Coswara cough, speech, and breathing datasets, respectively.

As indicated in Table 16 and Table 17, for the Coswara cough dataset (D2), among the base classifiers, A-BiLSTM and A-BiGRU obtained the ICBHI scores of 0.97 and 0.98, respectively, and showed better performances than those of the A-CRNN and CNN networks. The resulting ensemble model combining these base networks under an average probability scheme achieved an ICBHI score of 0.9710 for the identification of positive and negative COVID-19 cases using the coughing signals. In particular, owing to the small size of the Coswara cough dataset (D2) compared to that of ICBHI (D1), but with comparatively more clear audio coughing patterns, A-BiLSTM and A-BiGRU work better than other networks. 

Table 18 depicts the performances of the base networks with optimized learning settings for the Coswara speech dataset (D3). A-CRNN achieved the best ICBHI score of 0.9018, followed by A-BiLSTM and CNN with the ICBHI scores of 0.8916 and 0.888, respectively. In this Coswara speech dataset, the same speech was recorded across different subjects, and the disease-related voice symptoms are hidden inside the speech signals. Therefore, the identification of the respiratory abnormalities embedded in such speech signals is comparatively more difficult in comparison with using purely the coughing samples. The detection of subtle abnormal and healthy respiratory conditions requires the extraction of effective sequential temporal dynamics. The A-CRNN model equipped with 1D convolutional and BiLSTM layers is able to extract more diversified acoustic features than those obtained by other RNN or CNN models with homogenous layers (either BiLSTM/BiGRU or 1D convolutional layers). The PSO-based learning configuration selection and attention mechanisms further enhanced the feature learning capabilities of A-CRNN. Moreover, the diversified base networks with optimized distinctive learning settings also contributed to the superior performance of the resulting ensemble model for COVID-19 identification via speech, as evidenced in the confusion matrix shown in Table 19.

As indicated in existing studies [13,18], the detection of respiratory diseases using breathing data is a difficult task, in comparison with using coughing samples, which contain less obvious structural characteristics. As shown in Table 20, for the Coswara breathing dataset, A-BiLSTM and CNN achieved the best ICBHI scores, i.e., 0.9720 and 0.9030, respectively, for detecting COVID-19 using breathing instances. In particular, A-BiLSTM recognizes both positive and negative cases equally well, with high sensitivity and specificity scores. The least efficient model was A-BiGRU, with an ICBHI score of 0.8362. This is probably because of the comparatively simpler structures of the BiGRU/GRU layers embedded in A-BiGRU, in comparison with the BiLSTM/LSTM layers included in A-BiLSTM and A-CRNN. Table 21 shows the confusion matrix of the resulting ensemble model combining all four base networks for COVID-19 diagnosis using breathing samples.

### 4.3. Evaluation Results for the Combined Dataset D5 (ICBHI + Coswara Breathing) Using a Subject-Independent Split

As discussed earlier, we performed an 80–20 subject-independent train–test split for the combined ICBHI and Coswara breathing dataset (D5). Table 22 shows the detailed performance of the base and ensemble networks for the classification of a number of respiratory abnormalities against COVID-19 using the combined dataset. A-CRNN showed the best ICBHI score of 0.9555 for the 7-class lung condition diagnosis. Since A-BiLSTM and CNN extract purely homogenous features using monotonous layer structures, instead of diversified properties extracted using different types of layer topologies, they showed less competitive performances, with ICBHI scores of 0.8893 and 0.8867, respectively. Owing to its comparatively simpler network layer structures, A-BiGRU obtained the lowest ICBHI score of 0.8657. Moreover, because of the robust performance of the base networks, the ensemble model combining these distinctive base learners possesses sufficient complementary characteristics and achieved an ICBHI score of 0.9712. As indicated in the confusion matrix in Table 23, our ensemble model obtained high accuracy scores (0.8 or above) for the classification of nearly all lung conditions, except for URTI. 

### 4.4. Discussions

In this section, we further analyze the results of the ensemble networks for all the test datasets. The advantages and disadvantages of each proposed network are also elaborated.

#### 4.4.1. Result Analysis

We summarize the ensemble model performance for different datasets in Table 24. As indicated in Table 24, evaluated using four metric measurements, i.e., sensitivity, specificity, the ICBHI score, and the accuracy rate, the ensemble model performed exceptionally well across all five datasets. In particular, the ensemble model achieved ICBHI scores of 0.920 or above for all the test sets. For datasets D1 (ICBHI) and D5 (the combination of ICBHI and Coswara breathing), it achieved high ICBHI scores of 0.9766 and 0.9712, respectively, indicating a high level of robustness and reliability for multi-class lung abnormality classification. Especially, we employed D5, i.e., the customized combined dataset, with the intention to identify COVID-19 from other respiratory diseases such as COPD and bronchiolitis. The obtained high ICBHI score for this combined dataset, as shown in Table 24 and the confusion matrix in Table 23, indicates the great efficiency of the proposed model for diagnosing different lung diseases to help tackle the current pandemic. In other words, the empirical results indicate that it is indeed possible to make a reliable distinction between COVID-19 and other chronic and non-chronic conditions on a pathological level using the proposed ensemble model consisting of diverse distinctive base networks. 

With respect to the Coswara datasets, Table 24 shows that our ensemble model achieved the ICBHI scores of 0.971, 0.920, and 0.929, for the coughing (D2), speech (D3), and breathing (D4) subsets, respectively. The above results indicate that for coughing recordings from D2, which embeds structural characteristics of the sound signals, more effective MFCC features were extracted for audio classification. A possible explanation for this observation is that the nature of COVID-19, having major symptoms related to coughing, would thus likely result in providing more distinctive audio characteristics than those obtained from breathing or speech audio samples [49]. 

In addition to the reliable performance achieved using the coughing samples, the scores for speech and breathing recordings also indicate a high level of precision. The above results demonstrate that on a pathological level, a positive COVID-19 diagnosis can be classified to a high level of accuracy by all three channels. 

#### 4.4.2. Advantages and Disadvantages of the Proposed Base and Ensemble Networks

Based on the observations of the experimental results for all the datasets, all four base networks performed consistently well on each dataset, with the A-CRNN and A-BiLSTM models achieving the best mean ICBHI scores in most test cases, followed by those of the CNN model. A-BiGRU was the least effective network. We analyze the advantages and disadvantages of each base network and the ensemble model below. 

Both A-CRNN and A-BiLSTM models demonstrated consistent and reliable performances across the range of audio recordings. In particular, when tackling complex audio classification tasks (e.g., using multi-class ICBHI (D1) and the combined (D5) datasets), A-CRNN performed better than A-BiLSTM. The robustness of the A-CRNN model is attributed to the model topology and the efficient learning parameters. A-CRNN is composed of core layers such as 1D convolutional, BiLSTM, LSTM, and attention layers. The combination of 1D convolutional and BiLSTM layers is able to extract more diversified sequential cues than those obtained by A-BiLSTM and A-BiGRU consisting purely of the BiLSTM or BiGRU layers. In other words, the feature representations extracted by A-CRNN are more diversified in comparison with the homogenous attributes extracted by CNN, A-BiLSTM, and A-BiGRU. Moreover, the exploitation of the attention layer and PSO-optimized learning configurations in the A-CRNN model enables the network to extract more discriminative features to further enhance signal representations. The potential limitation of the network is that it requires comparatively larger training sets to demonstrate its full efficiency against other networks.

A-BiLSTM showed better efficiency than A-BiGRU and CNN in most test cases. The model applies bidirectional LSTM layers to extract temporal information from both forward and backward directions, which is further enhanced using the attention operations. In addition, the gating mechanisms in BiLSTM and LSTM layers are more complex than those embedded in the BiGRU and GRU layers. Specifically, a LSTM unit contains three gates, i.e., input, output, and forget gates, while a GRU only has two gates, i.e., reset and update gates [50]. Therefore, A-BiGRU has simpler layer structures with fewer parameters [51]. When tested using smaller datasets with comparatively more clear acoustic patterns, e.g., the Coswara cough dataset, both A-BiLSTM and A-BiGRU showed great efficiency in lung abnormality classification and achieved similar performances. However, when tackling complex multi-class audio classification tasks, e.g., using the ICBHI (D1) and combined (D5) datasets, A-BiLSTM shows better performance than those of A-BiGRU, owing to more complex gating mechanisms and layer topologies in A-BiLSTM. In addition, in comparison with A-CRNN, where both 1D convolutional and BiLSTM layers are used for feature extraction, the limitation of A-BiLSTM and A-BiGRU is the adoption of homogenous BiLSTM or BiGRU layers for feature learning. 

As the variant of 2D CNNs, a 1D CNN model with a set of 1D convolutional layers was also proposed in our studies. It performs scalar multiplication and addition operations in the 1D convolution for feature learning, with MaxPooling and AveragePooling layers used for feature dimension reduction. In comparison with a 2D CNN, the 1D CNN model can be applied to tackle audio signal classification using sequential audio feature inputs directly, without the requirement of converting waveforms to spectrograms as in 2D CNN to reduce costs. As a lightweight network, our 1D CNN model is compact and easier to train, with minimal computational costs. It showed efficiency in tackling classification tasks with data sparsity and achieved comparable performance across datasets, in comparison with the RNN models (e.g., A-BiLSTM). The limitation of the CNN model is the adoption of the same neuron type across different stages of the convolutional operations [52], which may limit its performance.

Moreover, an ensemble strategy was used to combine the results of the aforementioned networks. The four types of base networks embedding different learning mechanisms showed diverse learning behaviors to enhance ensemble diversity. On top of this, owing to PSO-based learning configuration optimization of different networks, the diversity and complementary characteristics of these base networks were further enhanced to boost ensemble performance. The empirical results indicate that the ensemble model combining four different optimized networks in a ‘majority voting’ mechanism for the classification of several audio datasets has proven to produce a reliable performance. The confusion matrices for all the test datasets shown in Section 4.1, Section 4.2 and Section 4.3 also indicate that on a class-by-class basis, the ensemble model can predict exceedingly confidently.

Table 25 illustrates the comparison with existing studies with respect to the ICBHI and Coswara cough datasets. Since different existing studies performed different classification tasks (e.g., binary, three-class, and six-class for the ICBHI dataset) and employed different training and test instances as well as evaluation methods (e.g., hold-out and cross-validation with random subject-dependent or subject-independent data splits), Table 25 serves as a loose performance comparison with related studies. 

For the ICBHI dataset, as indicated in Table 25, most existing studies, e.g., [13,22,23,24,26], categorized the six disease classes into healthy/unhealthy (two-class) or healthy/chronic/non-chronic (three-class) cases and performed binary or three-class predictions. In comparison with such classification tasks, our ensemble model performed a comparatively more challenging task for the identification of six respiratory abnormalities and achieved competitive performances. Moreover, most of the existing studies, such as [13,20,22,23], employed a random train–test split, instead of a subject-independent split, which may have audio clips from the same subjects allocated in both training and test sets, although the recordings were collected from different chest locations. In contrast, Zhang et al. [26] utilized a subject-independent split as those used in this research but their work conducted a three-class classification to identify healthy/chronic/non-chronic cases. García-Ordás et al. [24] also classified healthy/chronic/non-chronic cases using a 10-fold cross-validation with a random split. Such three-class lung condition detection is comparatively less challenging in comparison with the recognition of six different lung abnormalities. Similar to our studies, Wall et al. [20] performed a 6-class classification task but with a random 90–10 train–test split, instead of an 80–20 subject-independent split. 

In addition, most studies for the ICBHI dataset, such as Perna and Tagarelli [13], Wall et al. [20], and Wall et al. [22], adopted RNN models, e.g., LSTM, BiLSTM, or BiGRU, for lung abnormality detection, while a CNN with five convolutional layers and a CRNN model were used by García-Ordás et al. [24] and Zhang et al. [26], respectively. In comparison with these works, our ensemble model incorporates all the above different types of networks (i.e., BiLSTM, BiGRU, CNN, and CRNN), each with PSO-optimized learning hyper-parameters. Therefore, these base classifiers possess different learning behaviors and illustrate significantly complementary characteristics to enhance ensemble model performance. 

Furthermore, with respect to COVID-19 diagnosis using the Coswara cough dataset, the related work, i.e., Wall et al. [20], used a random 90–10 train–test split, while our studies employed a random 80–20 train–test split. As discussed above, our ensemble model integrating four types of base networks showed better performance than the single BiLSTM classifier used in [20]. 

In summary, our ensemble model embedding different optimized base networks showed sufficient capabilities and robustness for the identification of diverse lung abnormalities, in comparison with those of existing studies, and can be used as an effective alternative approach for lung abnormality classification.

## 5. Conclusions

In this research, we have proposed an ensemble model consisting of four deep networks, i.e., A-CRNN, A-BiLSTM, A-GRU, and CNN, for diverse lung abnormality classification using audio inputs. Our experimental results indicate that not only was audio classification of respiratory diseases possible, but also a high level of performance was attained. In addition, the ensemble majority voting scheme took advantage of diverse optimized base learners with different learning settings to deliver high levels of audio classification ICBHI scores. It could potentially be used as a competitive solution for clinical diagnosis. 

With the ICBHI scores ranging from 0.920 to 0.9766 over the five datasets, the ensemble model demonstrated great flexibility, consistency, and an overall exceedingly high level of performance in terms of sensitivity and specificity. Additionally, a novel dataset combining breathing recordings from ICBHI and Coswara datasets was also constructed with the aim of discovering if comparatively more common respiratory diseases could be distinguished from a positive COVID-19 diagnosis. It was found that not only could this be achieved, but again reliable metrics results were obtained using the proposed ensemble model. 

In future work, it would be worthwhile to expand the experiments to a wider range of medical audio datasets, potentially beyond respiratory diseases, to assess other conditions, such as neurological (e.g., Parkinson’s) diseases, since speech impairment is present in Parkinson’s and other neurological diseases. Additionally, it would also be worthwhile to deploy the proposed model to resource-constrained lightweight smartphone devices [53] to test its performance in real-world cases to help tackle Long-COVID-19 condition monitoring and rehabilitation.

## Figures and Tables

**Table 1:** The summary of existing studies for sound classification.

Related Studies	Methodologies	Novel Strategies
Choi et al. [9]	CRNN with four conv2d layers and two GRU layers for music classification using the Million Song dataset	-
Chen and Li [12]	(1) CNN-BiLSTM and 1D DNN for audio emotion classification, and (2) 1D DNN for lyrics emotion classification, using the Million Song dataset. (3) A stacking ensemble used to combine emotion classification results from both audio and text inputs.	(1) CNN-BiLSTM and 1D DNN for audio emotion classification, and (2) 1D DNN for lyrics emotion classification, using the Million Song dataset. (3) A stacking ensemble used to combine emotion classification results from both audio and text inputs.
Perna [23]	2D CNN	-
Perna and Tagarelli [13]	LSTM to train and classify the respiratory ICBHI dataset on both pathology and anomaly levels. The pathology-driven classification includes two tasks, i.e., binary (healthy/unhealthy) and 3-class (healthy/chronic/non-chronic) classification. On the other hand, for anomaly-driven diagnosis, a 4-class prediction is performed to detect normal/wheeze/crackle/both crackle and wheeze conditions.	Using different sliding window settings for data preparation
Pahar et al. [14]	Resnet50, LSTM, CNN, MLP, SVM, and LR for the classification of different lung abnormalities using the Coswara dataset and the SARS-CoV-2 South Africa (Sarcos) dataset.	-
Zhang et al. [18]	CRNN with attention mechanisms for environmental sound classification using ESC-10 and ESC-50 datasets.	CRNN with attention mechanisms
Wall et al. [20]	BiLSTM and BiGRU with attention mechanisms for respiratory and coughing sound classification	BiLSTM and BiGRU with attention mechanisms
Wall et al. [22]	BiLSTM for 2-class (health/unhealthy) respiratory sound classification	-
Zhang et al. [26]	An evolving ensemble of CRNNs for respiratory abnormality (healthy/chronic/non-chronic) classification, as well as heart sound and environmental sound classification.	Hyper-parameter fine-tuning using PSO (but for 3-class respiratory abnormality detection)
García-Ordás et al. [24]	2D CNN with two convolutional layers in combination with different data augmentation and oversampling techniques for respiratory abnormality classification	Adopting different oversampling techniques
Li et al. [27]	1D CNN with three convolutional layers for heart sound classification	-
Xiao et al. [28]	1D CNN with clique and transition blocks for heart sound classification	1D CNN with clique and transition blocks
Boddapati et al. [25]	AlexNet and GoogLeNet for environmental sound classification	-
Sait et al. [21]	Transfer learning based on Inception-v3 combined with MLP for COVID-19 diagnosis using breathing and chest X-ray image inputs	Transfer learning based on Inception-v3 combined with MLP for multimodal COVID-19 diagnosis
Zhang et al. [29]	2D CNN combined with sound mix-up	Sound mix-up for model training
This research	An evolving ensemble of A-CRNN, A-BiLSTM, A-BiGRU, and 1D CNN, with PSO-based hyper-parameter optimization	(1) CRNN, BiLSTM, and BiGRU with attention mechanisms (i.e., A-CRNN, A-BiLSTM, and A-BiGRU), as well as 1D CNN for audio classification. (2) PSO-based hyper-parameter tuning, and (3) an ensemble model combining the devised A-CRNN, A-BiLSTM, A-BiGRU, and 1D CNN.

**Table 2:** Dataset properties.

Dataset	Dataset Name	Class	No. of Files
D1	ICBHI	COPD	793
		Healthy	35
		Bronchiectasis	16
		Bronchiolitis	13
		URTI	23
		Pneumonia	37
		Asthma	1
		LRTI	2
D2	Coswara Cough	COVID-19 Positive	110
		COVID-19 Negative	107
D3	Coswara Speech	COVID-19 Positive	103
		COVID-19 Negative	104
D4	Coswara Breathing	COVID-19 Positive	101
		COVID-19 Negative	103
D5	ICBHI + Coswara Breathing	COPD	793
		Healthy	35
		Bronchiectasis	16
		Bronchiolitis	13
		URTI	23
		Pneumonia	37
		COVID-19	101

**Table 3:** The subject-independent train–test split for ICBHI used in our experiments.

	Training Set	Augmented Training Set	Test Set
Bronchiectasis	14	672	2
Bronchiolitis	7	672	6
URTI	16	672	7
Healthy	18	672	17
Pneumonia	30	660	7
COPD	648	648	145
Total	733	3996	184

**Table 4:** The subject-independent train–test split for the combined dataset (D5) based on ICBHI and Coswara breathing databases.

		Training Set	Augmented Training Set	Test Set
ICBHI (D1)	Bronchiectasis	14	672	2
	Bronchiolitis	7	672	6
	URTI	16	672	7
	Healthy	18	672	17
	Pneumonia	30	660	7
	COPD	648	648	145
Coswara Breathing (D4)	COVID-19	81	648	20
Total		814	4644	204

**Table 5:** Model 1—The proposed A-CRNN model architecture.

Layer#	Layer Description	Unit Setting	Kernel Size
L1	Conv1D	512	3
L2	Conv1D	256	3
L3	MaxPooling1D	N/A	N/A
L4	BiLSTM	512	N/A
L5	Attention Mechanism	N/A	N/A
L6	LSTM	256	N/A
L7	Dense	128	N/A
L8	FC Dense (Softmax)	Number of classes	N/A

**Table 6:** Model 2—The proposed A-BiLSTM network architecture.

Layer#	Layer Description	Unit Setting
L1	BiLSTM	512
L2	LSTM	256
L3	Attention Mechanism	N/A
L4	Dense	128
L5	Dropout	0.6
L6	Dense	64
L7	FC Dense (Softmax)	Number of classes

**Table 7:** Model 3—The proposed A-BiGRU network architecture.

Layer#	Layer Description	Unit Setting
L1	BiGRU	512
L2	GRU	256
L3	Attention Mechanism	N/A
L4	Dense	128
L5	Dropout	0.6
L6	Dense	64
L7	FC Dense (Softmax)	Number of classes

**Table 8:** Model 4—The proposed CNN architecture.

Layer#	Layer Description	Unit Setting	Kernel Size
L1	Conv1D	128	3
L2	Conv1D	128	3
L3	Conv1D	128	3
L4	MaxPooling1D	N/A	N/A
L5	Conv1D	256	3
L6	Conv1D	256	3
L7	Conv1D	256	3
L8	MaxPooling1D	N/A	N/A
L9	Conv1D	512	3
L10	Conv1D	512	1
L11	Conv1D	2	1
L12	GlobalAveragePooling1D	N/A	N/A
L13	Activation	N/A	N/A

**Table 9:** Optimized hyper-parameter settings with respect to D1.

Model	Hyper-Parameter	Setting
A-CRNN	Learning Rate	0.00159
	Batch Size	128
	Epoch	37
A-BiLSTM	Learning Rate	0.00095
	Batch Size	128
	Epoch	105
A-BiGRU	Learning Rate	0.00193
	Batch Size	128
	Epoch	45
CNN	Learning Rate	0.00019
	Batch Size	128
	Epoch	53

**Table 10:** Optimized hyper-parameter settings with respect to D2.

Model	Hyper-Parameter	Setting
A-CRNN	Learning Rate	0.000106
	Batch Size	64
	Epoch	26
A-BiLSTM	Learning Rate	0.000101
	Batch Size	64
	Epoch	15
A-BiGRU	Learning Rate	0.00909
	Batch Size	64
	Epoch	16
CNN	Learning Rate	0.00013
	Batch Size	64
	Epoch	23

**Table 11:** Optimized hyper-parameter settings with respect to D3.

Model	Hyper-Parameter	Setting
A-CRNN	Learning Rate	0.000143
	Batch Size	512
	Epoch	48
A-BiLSTM	Learning Rate	0.000099
	Batch Size	512
	Epoch	96
A-BiGRU	Learning Rate	0.00187
	Batch Size	512
	Epoch	33
CNN	Learning Rate	0.000122
	Batch Size	512
	Epoch	130

**Table 12:** Optimized hyper-parameter settings with respect to D4.

Model	Hyper-Parameter	Setting
A-CRNN	Learning Rate	0.000163
	Batch Size	512
	Epoch	48
A-BiLSTM	Learning Rate	0.000098
	Batch Size	512
	Epoch	96
A-BiGRU	Learning Rate	0.00083
	Batch Size	512
	Epoch	33
CNN	Learning Rate	0.000103
	Batch Size	512
	Epoch	130

**Table 13:** Optimized hyper-parameter settings with respect to D5.

Model	Hyper-Parameter	Setting
A-CRNN	Learning Rate	0.000157
	Batch Size	128
	Epoch	42
A-BiLSTM	Learning Rate	0.000197
	Batch Size	128
	Epoch	30
A-BiGRU	Learning Rate	0.00192
	Batch Size	128
	Epoch	38
CNN	Learning Rate	0.000083
	Batch Size	128
	Epoch	43

**Table 14:** Results of base and ensemble models using a subject-independent train–test split for D1, i.e., the ICBHI dataset.

Models	Sensitivity	Specificity	ICBHI Score	Accuracy
A-CRNN	0.8947	1	0.9474	0.8989
A-BiLSTM	0.8947	0.8571	0.8759	0.8933
A-BiGRU	0.8655	0.8571	0.8613	0.8652
CNN	0.883	0.8571	0.8701	0.882
Ensemble	0.9532	1	0.9766	0.9551

**Table 15:** Confusion matrix of the proposed ensemble model for D1, i.e., the ICBHI dataset.

	Bronchiectasis	Bronchiolitis	COPD	Healthy	Pneumonia	URTI
Bronchiectasis	1	0	0	0	0	0
Bronchiolitis	0	1	0	0	0	0
COPD	0.0261	0	0.9673	0.0065	0	0
Healthy	0	0	0	1	0	0
Pneumonia	0	0	0.2	0	0.8	0
URTI	0	0	0	0.4	0	0.6

**Table 16:** Results of base and ensemble models for D2, i.e., the Coswara cough dataset.

Models	Sensitivity	Specificity	ICBHI Score	Accuracy
A-CRNN	0.9231	0.8846	0.9038	0.9060
A-BiLSTM	1	0.9391	0.9700	0.9754
A-BiGRU	1	0.9600	0.9800	0.9825
CNN	0.9524	0.9077	0.9300	0.9297
Ensemble	1	0.9420	0.9710	0.9750

**Table 17:** Confusion matrix of the proposed ensemble model for D2, i.e., the Coswara cough dataset.

	Positive	Negative
Positive	1	0
Negative	0.058	0.942

**Table 18:** Results of base and ensemble models for D3, i.e., the Coswara speech dataset.

Models	Sensitivity	Specificity	ICBHI Score	Accuracy
A-CRNN	0.9289	0.8747	0.9018	0.9023
A-BiLSTM	0.9422	0.8410	0.8916	0.8894
A-BiGRU	0.8344	0.8258	0.8300	0.8304
CNN	0.8965	0.8795	0.8880	0.8881
Ensemble	0.9480	0.8920	0.9200	0.9240

**Table 19:** Confusion matrix of the proposed ensemble model for D3, i.e., the Coswara speech dataset.

	Positive	Negative
Positive	0.9480	0.0520
Negative	0.1080	0.8920

**Table 20:** Results of base and ensemble models for D4, i.e., the Coswara breathing dataset.

Models	Sensitivity	Specificity	ICBHI Score	Accuracy
A-CRNN	0.9073	0.8746	0.8909	0.8936
A-BiLSTM	0.9909	0.9530	0.9720	0.9724
A-BiGRU	0.8654	0.8069	0.8362	0.8320
CNN	0.9497	0.8562	0.9030	0.9066
Ensemble	0.9810	0.8770	0.9290	0.9300

**Table 21:** Confusion matrix of the proposed ensemble model for D4, i.e., the Coswara breathing dataset.

	Positive	Negative
Positive	0.9810	0.019
Negative	0.1230	0.8770

**Table 22:** Results of the base and ensemble models using a subject-independent train–test split for D5, i.e., the combination of ICBHI and Coswara breathing datasets.

Models	Sensitivity	Specificity	ICBHI Score	Accuracy
A-CRNN	0.911	1	0.9555	0.9141
A-BiLSTM	0.9215	0.8571	0.8893	0.9192
A-BiGRU	0.8743	0.8571	0.8657	0.8737
CNN	0.9162	0.8571	0.8867	0.9141
Ensemble	0.9424	1	0.9712	0.9444

**Table 23:** Confusion matrix of the proposed ensemble model for D5, i.e., the combination of ICBHI and Coswara breathing datasets.

	Bronchiectasis	Bronchiolitis	COPD	Healthy	Pneumonia	URTI	COVID-19
Bronchiectasis	1	0	0	0	0	0	0
Bronchiolitis	0	0.8333	0	0.1667	0	0	0
COPD	0.0261	0	0.9542	0.0196	0	0	0
Healthy	0	0	0	1	0	0	0
Pneumonia	0	0	0	0	0.8	0.2	0
URTI	0	0	0	0.4	0	0.6	0
COVID-19	0	0	0	0	0	0	1

**Table 24:** Ensemble results for the five test datasets.

Dataset	Sensitivity	Specificity	ICBHI Score	Accuracy
D1	0.9532	1	0.9766	0.9551
D2	1	0.942	0.971	0.975
D3	0.948	0.892	0.920	0.924
D4	0.981	0.877	0.929	0.930
D5	0.9424	1	0.9712	0.9444

**Table 25:** Performance comparison with existing studies.

	Existing Studies	Methodology	No. of Classes	Evaluation Strategies	Results
ICBHI	Wall et al. [20]	BiLSTM with attention mechanisms	6	90–10 (random)	Accuracy rate—0.962
	Zhang et al. [26]	An evolving ensemble of CRNNs	3 (healthy, chronic, and non-chronic)	80–20 (subject-independent)	ICBHI score—0.9803
	Wall et al. [22]	BiLSTM	2 (healthy and unhealthy)	80–20 (random)	ICBHI score—0.957
	Perna [23]	2D CNN	3 (healthy, chronic, and non-chronic)	80–20 (random)	ICBHI score—0.83
	Perna and Tagarelli [13]	LSTM with 50% overlapping between windows	3 (healthy, chronic, and non-chronic)	80–20 (random)	ICBHI score—0.9
	Perna and Tagarelli [13]	LSTM without overlapping	3 (healthy, chronic, and non-chronic)	80–20 (random)	ICBHI score—0.89
	García-Ordás et al. [24]	2D CNN with Synthetic Minority Oversampling Technique	3 (healthy, chronic, and non-chronic)	10-fold (random)	ICBHI score—0.558
	García-Ordás et al. [24]	2D CNN with Adaptive Synthetic Sampling Method	3 (healthy, crohnic, and non-crohnic)	10-fold (random)	ICBHI score—0.911
	García-Ordás et al. [24]	2D CNN with dataset weighted	3 (healthy, chronic, and non-chronic)	10-fold (random)	ICBHI score—0.476
	This research	Ensemble of optimized A-CRNN, A-BiLSTM, A-BiGRU, and 1D CNN	6	80–20 (subject-independent)	ICBHI score—0.9766 Acccuracy rate—0.9551
Coswara (cough)	Wall et al. [20]	BiLSTM with attention mechanisms	2	90–10 (random)	Accuracy rate—0.968
	This research	Ensemble of optimized A-CRNN, A-BiLSTM, A-BiGRU, and 1D CNN	2	80–20 (random)	ICBHI score—0.971 Acccuracy rate—0.975

## Data Availability

The datasets employed in this study are publicly available at the sites of ICBHI 2017 Challenge (https://bhichallenge.med.auth.gr/ICBHI_2017_Challenge, accessed on 1 April 2022) and the Coswara Database (https://github.com/iiscleap/Coswara-Data, accessed on 1 April 2022).
